# Neonatal brain injuries in England: population-based incidence derived from routinely recorded clinical data held in the National Neonatal Research Database

**DOI:** 10.1136/archdischild-2017-313707

**Published:** 2017-10-22

**Authors:** Chris Gale, Yevgeniy Statnikov, Sena Jawad, Sabita N Uthaya, Neena Modi

**Affiliations:** 1 Section of Neonatal Medicine, Department of Medicine, Imperial College London, Chelsea and Westminster Hospital campus, London, UK; 2 Society and College of Radiographers, London, London, UK

**Keywords:** nnrd, brain injuries, infant, newborn, electronic health records, intensive care, neonatal

## Abstract

**Objective:**

In 2015, the Department of Health in England announced an ambition to reduce ‘brain injuries occurring during or soon after birth’. We describe the development of a pragmatic case definition and present annual incidence rates.

**Design:**

Retrospective cohort study using data held in the National Neonatal Research Database (NNRD) extracted from neonatal electronic patient records from all National Health Service (NHS) neonatal units in England, Wales and Scotland. In 2010–2011, population coverage in the NNRD was incomplete, hence rate estimates are presented as a range; from 2012, population coverage is complete, and rates (95% CIs) are presented. Rates are per 1000 live births.

**Setting:**

NHS neonatal units in England.

**Patients:**

Infants admitted for neonatal care; denominator: live births in England.

**Main outcome measure:**

‘Brain injuries occurring at or soon after birth’ defined as infants with seizures, hypoxic-ischaemic encephalopathy, stroke, intracranial haemorrhage, central nervous system infection and kernicterus and preterm infants with cystic periventricular leucomalacia.

**Results:**

In 2010, the lower estimate of the rate of ‘Brain injuries occurring at or soon after birth’ in England was 4.53 and the upper estimate was 5.19; in 2015, the rate was 5.14 (4.97, 5.32). For preterm infants, the population incidence in 2015 was 25.88 (24.51, 27.33) and 3.47 (3.33, 3.62) for term infants. Hypoxic-ischaemic encephalopathy was the largest contributor to term brain injury, and intraventricular/periventricular haemorrhage was the largest contributor to preterm brain injury.

**Conclusions:**

Annual incidence rates for brain injuries can be estimated from data held in the NNRD; rates for individual conditions are consistent with published rates. Routinely recorded clinical data can be used for national surveillance, offering efficiencies over traditional approaches.

**What is already known on this topic?**

- Conditions that lead to brain injury around the time of birth are leading causes of mortality and morbidity.
- Reducing ‘brain injuries occurring during or soon after birth’ in England is part of a wider national maternity ambition driven by the Department of Health.
- There are limited population data on the incidence of many of the conditions that lead to brain injury around the time of birth.

**What this study adds?**

- An expert working group has constructed a pragmatic, working definition for ‘brain injuries occurring during or soon after birth’.
- Annual population incidence data for conditions leading to neonatal brain injury can be estimated from routinely recorded data held in the National Neonatal Research Database (NNRD).
- Annual population incidence rates in England for conditions leading to neonatal brain injury, calculated using data from the NNRD, are in agreement with limited published rates from other developed healthcare settings.

## Introduction

Conditions that lead to brain injury around the time of birth,^1^ such as hypoxia-ischaemia, stroke and intracranial haemorrhage, are leading causes of neonatal mortality^2^ and life-long morbidities including some forms of cerebral palsy.^3^

In November 2015, the UK Secretary of State for Health announced a national ambition to reduce ‘*brain injuries occurring during or soon after birth*’ by 20% by 2020 and halve them by 2030. This was part of a wider national maternity ambition to similarly reduce stillbirths and maternal and neonatal deaths.^4^

The Department of Health commissioned the Neonatal Data Analysis Unit at Imperial College London to estimate annual figures for ‘*brain injuries occurring during or soon after birth*’ from routinely recorded clinical data held in the National Neonatal Research Database (NNRD). The NNRD, a national resource, was chosen by the Department of Health, because it is the only data source that met the requirements for coverage, including estimation of retrospective rates and trends over time, frequent reporting and ability to identify brain injury without need for information in childhood.

Here we describe the development of a working definition for ‘*brain injuries occurring during or soon after birth*’ and report national incidence rates for England over the period 2010–2015. We also report national incidence rates for individual conditions included in the working definition.

## Methods

### Defining brain injuries occurring at or soon after birth

Unlike stillbirth or neonatal death, the term ‘*brain injuries occurring during or soon after birth*’ does not have an agreed or standardised definition. An expert group was therefore convened by the Department of Health to determine a working definition for the national maternity ambition. The group met at Richmond House, London, on 17 January 2017; experts in neonatal and perinatal brain injury, health professionals and representatives of government and other National Health Service (NHS) organisations were invited. Attendees and their affiliations are listed in online supplementary data 1. The remit of the expert group was to reach consensus on a pragmatic working definition for ‘*brain injuries occurring during or soon after birth*’ and describe the following components of such a definition: scope (including the gestational age of infants and the timing of injury), conditions to be included, data items to be included and the denominator to be used to calculate incidence. The final working definition for ‘*brain injuries occurring during or soon after birth*’ agreed by the expert group for the purposes of the national maternity ambition is described in box; the corresponding NNRD data items can be found in online supplementary data 2. The definition represents a pragmatic approach to identifying infants likely to have sustained brain injury, using signs of acute neurological dysfunction and conditions known to be causally related.

Box **Consensus definition of ‘*brain injuries occurring at or soon after birth*’**

Brain injuries occurring at or soon after birth: final working definition for the Department of Health national maternity ambition.

Population: all neonatal unit admissions.

Time period after birth: all brain injuries detected during neonatal unit stay.

Conditions to be included:

- seizures: all infants
- intracranial haemorrhage, perinatal stroke, hypoxic-ischaemic encephalopathy, central nervous system infection, kernicterus (bilirubin encephalopathy): all infants
- white matter disease (periventricular leukomalacia): preterm infants only.

Denominator: all live births.

Exclusions: congenital encephalopathies (including inborn errors of metabolism), congenital infections and congenital brain abnormalities: a consensus decision was made to present data before and after exclusion of infants with seizures, who also had a diagnosis of a condition indicating that brain injury had occurred prior to birth.

The data items within the National Neonatal Research Database that constitute the final working definition of ‘brain injuries occurring at or soon after birth’ can be found in online supplementary data 2.

10.1136/archdischild-2017-313707.supp1 Supplementary file 1

10.1136/archdischild-2017-313707.supp2 Supplementary file 2

## Data sources

Case (numerator) data were extracted from the NNRD. The NNRD is a clinical dataset (The National Neonatal Data Set) within the NHS Data Dictionary. Details of all data items are searchable at the following web page: http://www.datadictionary.nhs.uk/data_dictionary/messages/clinical_data_sets/data_sets/national_neonatal_data_set/national_neonatal_data_set_-_episodic_and_daily_care_fr.asp?shownav=1.

In the UK, summary electronic patient data are entered on all admissions to NHS neonatal units in England, Wales and Scotland. Approximately 450 predefined data items that form the National Neonatal Data Set, an approved NHS Information Standard,^5^ are extracted at regular intervals from the real-time electronic platform and held in a unique national resource,^5^ the NNRD. Data in the NNRD have undergone detailed cleaning to identify, for example, duplicates, out of range values, internal inconsistencies and other potentially erroneous entries. In addition to these internal processes, feedback quality assurance checks are undertaken with clinicians for key items. Data in the NNRD are merged across multiple patient episodes to create a single record for each infant.^6^ The NNRD holds data from approximately 90% of English NHS neonatal units from 2010 and from 100% of English NHS neonatal units from 2012 onwards. The NNRD only holds data on infants admitted for neonatal care; no data are held on infants in the neonatal period who are receiving care on postnatal or paediatric wards.

The specific National Neonatal Data Set items used to define ‘*brain injuries occurring during or soon after birth*’ for the national maternity ambition are described in online supplementary data 2. Denominator data, total live births in England, were obtained from the Office for National Statistics (ONS),^7^ as were total live births by gestational age.^8 9^

To calculate national rates of ‘*brain injuries occurring during or soon after birth*’, infants were categorised as a case of ‘brain injury’ if they were recorded as having any of the conditions described in box; therefore, an infant with both seizures and cystic periventricular leucomalacia was counted once. When calculating rates for the individual conditions included in ‘*brain injuries occurring during or soon after birth*’, infants were categorised for each condition separately; therefore, an infant with both seizures and cystic periventricular leukomalacia was counted as a case for both conditions.

## Statistical methods

For the period 2012–2015, when the NNRD had population coverage for neonatal admissions in England, brain injury data are presented as absolute counts and rates with 95% CIs.

In 2010 and 2011, approximately 90% of NHS neonatal units in England contributed data to the NNRD; to account for missing data, national rates of brain injuries in England for 2010 and 2011 were estimated. These rates are hence more uncertain than rates reported for subsequent years. The Department of Health required rates to be estimated from 2010 to allow reporting of ‘*brain injuries occurring during or soon after birth*’ alongside other measures included in the national maternity ambition, namely stillbirths, maternal deaths and neonatal deaths.

In 2010 and 2011, the NNRD held data from contributing neonatal units and data on total births in England (from ONS), but the number of infants admitted to neonatal units that did not contribute to the NNRD and the number of total live births in maternity units attached to these non-contributing neonatal units were not known. To account for this, admissions for 2010 and 2011 were estimated using data on annual neonatal unit admissions from 2012 to 2015, when the NNRD had complete coverage of neonatal units in England. There was an upward trend in the number of neonatal unit admissions from 2012 to 2015, suggesting that the admissions in 2010 and 2011 would have been lower than the later years. Lower and upper inflations in the number of admissions were estimated based on the most extreme yearly increases seen in 2012–2015, assuming that the admissions in 2010 and 2011 would be as, or less, extreme than these. The SD of the proportion of brain injuries to admissions was estimated using actual data from the NNRD; the number of brain injuries on the lower increase in admissions was inflated by two SD less than the actual brain injury rate for the lower estimate and by an increase in two SD more than the actual brain injury for the upper estimate. This method gave more of an overestimate for the upper estimate than the lower estimate. Inflated rates for brain injuries were then calculated by taking the lower and higher estimates of brain injuries as a proportion of the total number of live births, under the assumption that the brain injury rate among the missing infants would not differ from the calculated rate. Rates of brain injuries for 2010 and 2011 are presented as ranges that reflect these lower and higher calculated estimates.

For all years, rates were stratified by gestational age with preterm infants defined as those born at <37 weeks and term infants those born at ≥37 weeks.

## Results

The number of infants admitted to neonatal units contributing data to the NNRD increased from 64 375 in 2010 to 88 785 in 2015; the annual rate of brain injuries after exclusions in England in 2010 was between 4.53 and 5.19 per 1000 live births; in 2015, the rate was 5.14 (95% CI 4.97 to 5.32) per 1000 live births (table 1); annual data for 2010–2015 are presented table 1.

**Table 1:** Infants in England (all gestational ages) with a diagnosis of brain injury, before and after exclusion of conditions leading to brain injury prior to birth

Year	Infants recorded in the NNRD	Live births in England	Infants with brain injury, before exclusions	Exclusions	Infants with brain injury, after exclusions	Infants with brain injury adjusted for incomplete NNRD coverage	Infants with brain injury adjusted for incomplete NNRD coverage, after exclusions	Rate of brain injuries per 1000 live births	Brain injuries per 1000 live births, after exclusions (95% CI)
2010	64 375	687 007	3011	45	2966	3160 to 3619	3113 to 3566	4.60 to 5.27	4.53 to 5.19
2011	72 678	688 120	3377	46	3331	3434 to 3630	3387 to 3581	4.99 to 5.28	4.93 to 5.20
2012	78 952	694 241	3404	45	3359	Not adjusted	Not adjusted	4.90 (4.47 to 5.07)	4.84 (4.68 to 5.00)
2013	80 199	664 517	3393	35	3358	Not adjusted	Not adjusted	5.11 (4.94 to 5.28)	5.05 (4.89 to 5.23)
2014	84 981	661 496	3558	30	3528	Not adjusted	Not adjusted	5.38 (5.20 to 5.56)	5.33 (5.16 to 5.51)
2015	88 785	664 399	3445	27	3418	Not adjusted	Not adjusted	5.19 (5.01 to 5.36)	5.14 (4.97 to 5.32)

Data for 2010 and 2011 are adjusted to account for the incomplete coverage of the NNRD during those years; from 2012 onwards, the NNRD has complete population coverage of neonatal admissions in England so no adjustment was necessary, and data are presented as a rate (95% CI).

NNRD, National Neonatal Research Database.

The annual rate of brain injuries among term infants (≥37 gestational weeks) in England in 2015 was 3.47 (95% CI 3.33 to 3.62) per 1000 live term births; data for term infants born over the period 2010–2015 are presented in table 2. The annual rate of brain injuries among preterm infants (<37 gestational weeks) in England in 2015 was 25.88 (95% CI 24.51 to 27.33) per 1000 live preterm births; data for preterm infants between 2010 and 2015 can be found in table 3.

**Table 2:** Term (≥37 gestational weeks) infants in England with a diagnosis of brain injury, before and after exclusion of conditions leading to brain injury prior to birth

Year	Term infants recorded in the NNRD	Term live births in England	Term infants with brain injury, before exclusions	Term infants with brain injury adjusted for incomplete NNRD coverage	Term infants with brain injury, after adjustment and exclusions	Rate of brain injuries per 1000 term live births, after exclusions (95% CI)
2010	35 415	627 357	1830	1979 to 2218	1949 to 2186	3.11 to 3.48
2011	41 429	630 419	2126	2213 to 2285	2179 to 2249	3.46 to 3.57
2012	46 200	640 787	2109	Not adjusted	2074	3.24 (3.10 to 3.38)
2013	47 935	612 816	2130	Not adjusted	2105	3.43 (3.29 to 3.58)
2014	51 945	607 972	2215	Not adjusted	2189	3.60 (3.45 to 3.75)
2015	55 045	609 076	2136	Not adjusted	2116	3.47 (3.33 to 3.62)

Data for 2010 and 2011 are adjusted to account for the incomplete coverage of the NNRD during these years; from 2012 onwards, the NNRD has complete population coverage of neonatal admissions in England so no adjustment was necessary, and data are presented as a rate (95% CI).

NNRD, National Neonatal Research Database.

**Table 3:** Preterm (<37 gestational weeks) infants in England with a diagnosis of brain injury, before and after exclusion of conditions leading to brain injury prior to birth

Year	Preterm infants recorded in the NNRD	Preterm live births in England	Preterm infants with brain injury, before exclusions	Preterm infants with brain injury adjusted for incomplete NNRD coverage	Preterm infants with brain injury after adjustment and exclusions	Rate of brain injuries per 1000 preterm live births, after exclusions (95% CI)
2010	28 960	43 928	1181	1273 to 1310	1254 to 1290	28.54 to 29.37
2011	31 249	44 547	1251	1281 to 1298	1268 to 1284	28.47 to 28.83
2012	32 752	49 949	1295	Not adjusted	1285	25.73 (24.36 to 27.17)
2013	32 264	48 844	1263	Not adjusted	1253	25.65 (24.27 to 27.11)
2014	33 036	49 379	1343	Not adjusted	1339	27.12 (25.70 to 28.61)
2015	33 740	50 308	1309	Not adjusted	1302	25.88 (24.51 to 27.33)

Data for 2010 and 2011 are adjusted to account for the incomplete coverage of the NNRD during these years; from 2012 onwards, the NNRD has complete population coverage of neonatal admissions in England so no adjustment was necessary, and data are presented as a rate (95% CI).

NNRD, National Neonatal Research Database.

The consensus opinion was that the following individual conditions make up the working definition of ‘*brain injuries occurring during or soon after birth*’: neonatal seizures, intracranial haemorrhage (including intraventricular/periventricular haemorrhage), perinatal/neonatal stroke, hypoxic-ischaemic encephalopathy (HIE), central nervous system infection, bilirubin encephalopathy and, among preterm infants only, cystic periventricular leukomalacia. Annual numbers and population rates for these conditions calculated from NNRD data are presented in table 4. Data are from 2012 onwards when all NHS neonatal units contributed to the NNRD.

**Table 4:** Infants in England with conditions leading to brain injury at or soon after birth

Condition		Year			
		2012	2013	2014	2015
Seizures	Number of cases	1445	1432	1360	1249
	Rate per 1000 live births (95% CI)	2.1 (2.0 to 2.1)	2.2 (2.1 to 2.3)	2.1 (2.0 to 2.2)	1.9 (1.8 to 2.0)
	Number of term cases	1065	1036	1009	919
	Number of preterm cases	378	396	351	330
Intracranial haemorrhage	Number of cases	754	677	689	726
	Rate per 1000 live births (95% CI)	1.1 (1.0 to 1.2)	1.0 (0.9 to 1.1)	1.0 (1.0 to 1.1)	1.1 (1.0 to 1.2)
	Number of term cases	110	94	104	117
	Rate per 10 000 term births (95% CI)	1.7 (1.4 to 2.1)	1.5 (1.3 to 1.9)	1.7 (1.4 to 2.1)	1.9 (1.6 to 2.3)
	Number of preterm cases	644	583	585	609
	Severe P/IVH <32 weeks gestation	483	445	468	452
	Rate of severe P/IVH per 1000 live births <32 weeks (95% CI)	60.4 (55.2 to 66.0)	57.7 (52.6 to 63.4)	61.1 (55.8 to 66.9)	58.3 (53.1 to 63.9)
Perinatal/neonatal stroke	Number of cases	77	100	88	90
	Rate per 1000 live births (95% CI)	0.11 (0.09 to 0.14)	0.15 (0.12 to 0.18)	0.13 (0.11 to 0.16)	0.14 (0.11 to 0.17)
	Number of term cases	64	78	72	76
	Number of preterm cases	13	22	16	14
Hypoxic-ischaemic encephalopathy	Number of cases	1674	1674	1824	1742
	Rate per 1000 live births (95% CI)	2.4 (2.3 to 2.5)	2.5 (2.4 to 2.6)	2.8 (2.6 to 2.9)	2.6 (2.5 to 2.8)
	Number of term cases	1409	1401	1480	1417
	Number of preterm cases	265	273	344	325
Central nervous system infection	Number of cases	353	422	504	465
	Rate per 1000 live births (95% CI)	0.51 (0.46 to 0.56)	0.64 (0.58 to 0.70)	0.76 (0.70 to 0.83)	0.70 (0.64 to 0.77)
	Number of term cases	188	266	284	277
	Number of preterm cases	165	156	220	188
Bilirubin encephalopathy	Number of cases	8	7	2	4
	Rate per 100 000 live births (95% CI)	1.2 (0.6 to 2.3)	1.1 (0.5 to 2.2)	0.3 (0.1 to 1.2)	0.6 (0.2 to 1.6)
	Number of term cases	6	5	2	4
	Number of preterm cases	2	2	0	0
Cystic periventricular leucomalacia	Number of preterm cases	199	175	171	184
	Rate per 1000 live births (95% CI)	0.3 (0.3 to 0.3)	0.3 (0.2 to 0.3)	0.3 (0.2 to 0.3)	0.3 (0.2 to 0.3)
	Number of cases at <34 weeks gestation	186	175	157	176
	Rate per 1000 live births <34 weeks gestation (95% CI)	12.7 (11.0 to 14.7)	12.5 (10.7 to 14.4)	11.3 (9.7 to 13.3)	12.4 (10.7 to 14.4)

Infants can be diagnosed with more than one condition so the sum of conditions for each year will not match data given in tables 1–3; P/IVH, periventricular/intraventricular haemorrhage.

## Discussion

We present national incidence rates for ‘*brain injuries occurring during or soon after birth*’ based on pragmatic definitions, agreed on by a multidisciplinary expert group, from data held in the NNRD relating to infants admitted to neonatal units in England. To our knowledge, this work is unique in describing and calculating a summary measure of conditions leading to perinatal and neonatal brain injury at a national population level. We demonstrate that the national incidence of ‘*brain injuries occurring during or soon after birth*’ in England has remained relatively consistent over the period 2010 to 2015. This analysis was commissioned by the Department of Health in England as part of the National Maternity Ambition, which aims to reduce maternal and neonatal deaths, stillbirths and brain injuries. The longitudinal assessment of the incidence of brain injuries, using standardised definitions and without the burden of additional data collection, should facilitate benchmarking, quality improvement and research aiming to reduce these conditions and the associated life-long medical and neurocognitive burdens.

To our best knowledge, this is the largest and most complete attempt to define population incidence figures for neonatal and perinatal brain injuries. A strength of our study was the consistent application of a predefined case definition across a globally unique population-level repository of point-of-care clinician-entered patient data—the NNRD. As there is no agreed or established definition for ‘*brain injuries that occur at or soon after birth*’, and as brain injury in infants can manifest in diverse ways and have many causes,^1^ an expert panel was convened to establish a pragmatic working definition. The definition agreed represents a compromise between two requirements set by the Department of Health: first, to capture infants with perinatal or neonatal brain injury reliably and completely, and second, to use existing data and report rates retrospectively back to 2010. As a result, the definition includes both signs of acute neurological dysfunction, seizures, and conditions, such as HIE, that are markers of potential brain injury rather than definitive evidence that such injury has occurred. Clear evidence of injury often only emerges as the child grows older, hence an accurate measure would require complete long-term follow-up and assessment in childhood. A further limitation is that data were only available from infants admitted for neonatal care, therefore any infants managed on postnatal or paediatric wards would not be captured. However, such instances are likely to be few in number. It is also important to acknowledge that diagnostic certainty at the point of data entry varies between conditions; neonatal seizures and the reasons for them can be difficult to diagnose in the neonatal period, and this may result in underestimation of the rate for this condition. The clinical experience of medical staff completing the summary electronic patient record may vary between neonatal units, which may impact data quality, particularly in 2010–2011, when the system was being introduced to new neonatal units. We endeavoured to make clear the limitations inherent in the data, specifically the assumptions required to estimate national rates for 2010 and 2011.

The novelty of the measure ‘*brain injuries that occur at or soon after birth*’, and the source of the data, the NNRD which is formed from routinely recorded clinical information, makes it necessary to consider how incidence rates of individual conditions we report compare with other published data. The annual incidence rates for moderate and severe HIE of between 2.4 and 2.8 per 1000 live births are consistent with other reported rates of neonatal encephalopathy of between 0.77 and 3.8 per 1000 live births in low neonatal mortality regions, such as the UK and the USA.^10^ When considering neonatal intracranial haemorrhage, published data commonly reported incidence separately for term and preterm infants. A 30-year-old, single-centre study from the USA reported a regional incidence of 2.7 per 10 000 live births for symptomatic intracranial haemorrhage in term infants,^11^ which is comparable with the population incidence of 1.5–1.9 per 10 000 term births that we report. For preterm infants born at 22–31 weeks gestational age, comparable population-level incidence data for intraventricular/periventricular haemorrhage from the national French EPIPAGE cohort are 3.8% for grade 3% and 3.3% for grade 4 intra/periventricular haemorrhage.^12^ In the same gestational age band, we report annual incidence rates between 5.8% and 6.1% for a composite including grades 3 and 4 intraventricular/periventricular haemorrhage. We report annual incidence rates for neonatal or perinatal stroke of between 0.11 and 0.15 per 1000 live births, which is similar to the estimated minimum incidence rate of 0.10 per 1000 live births reported by a prospective, population-based study from Canada.^13^ The annual incidence of neonatal central nervous system infection we report, of 0.51–0.76 per 1000 live births, is similarly in agreement with the population-level incidence rate for neonatal meningitis in England and Wales of 0.39 per 1000 live births (1996–1997) reported by the British Paediatric Surveillance Unit (BPSU).^14^ Similarly, annual rates of bilirubin encephalopathy reported here of between 0.3 and 1.2 per 100 000 live births are comparable with BPSU population surveillance rates of 0.9 per 1 00 000 live births (2003–2005).^15^ EPIPAGE 2 (2011) reports an incidence among 23–34 gestational week infants of 1.8% for cystic periventricular leukomalacia^16^; in the same gestational age group, we report annual rates of between 1.13% and 1.27%. Finally, population-based studies of neonatal seizures over the last 30 years report incidence rates between 1.8^17^ and 3.5^18^ per 1000 live births, results that are again in keeping with our annual rates of 1.9–2.2 per 1000 live births.

In conclusion, we provide national estimates for ‘*brain injuries occurring at or soon after birth*’ for England over the period 2010–2015 using data held in a national database derived from point-of-care, clinician professional-entered electronic patient records. Our estimates are in broad agreement with other published population-based rates from stand-alone studies conducted in similar neonatal care settings, using data recorded specifically for each study. This supports the validity of using data held in the NNRD for initiatives such as the Department of Health national maternity ambition. This approach is cost and time efficient, less burdensome for health professionals and represents a positive evolution away from the former approach of bespoke duplicative data collections for each specific purpose.
