# Correction: *Neonatal brain injuries in England: population-based incidence derived from routinely recorded clinical data held in the National Neonatal Research Database*

**DOI:** 10.1136/archdischild-2017-313707corr1

**Published:** 2021-03-09

Gale C, Statnikov Y, Jawad S, *et al*. Neonatal brain injuries in England: population-based incidence derived from routinely recorded clinical data held in the National Neonatal Research Database. *Arch Dis Childhood – Fetal and Neonatal Edition* 2018;103:F301–6.

The authors have identified an error in data extraction that resulted in the following: (1) the inclusion of babies with Grade 1 HIE who did not receive therapeutic hypothermia; (2) the exclusion of babies with central nervous system infection after 48 hours after birth. A data transcription error resulted in incorrect numbers of exclusions. Therefore the following sections are corrected as outlined below.

## Abstract results

The results section of the Abstract should be as follows: ‘In 2010, the lower estimate of the rate of ‘Brain injuries occurring at or soon after birth’ in England was 3.99 and the upper estimate was 4.66; in 2015, the rate was 4.52 (4.36 to 4.68). For preterm infants, the population incidence in 2015 was 24.45 (23.12 to 25.85) and for term infants 2.91 (2.78 to 3.05). Hypoxic ischaemic encephalopathy was the largest contributor to term brain injury and intraventricular/periventricular haemorrhage was the largest contributor to preterm brain injury.’

## Results

The first and second paragraph of the Results section of the manuscript are corrected to ‘The number of infants admitted to neonatal units contributing data to the NNRD increased from 64 375 in 2010 to 88 785 in 2015; the annual rate of brain injuries after exclusions in England in 2010 was between 3.87 and 4.44 per 1000 live births; in 2015 to the rate was 4.52 (95% CI 4.36 to 4.68) per 1000 live births (table 1); annual data for 2010 to 2015 are presented table 1.’

**Table 1:** Infants in England (all gestational ages) with a diagnosis of brain injury to before and after exclusion of conditions leading to brain injury prior to birth; data for 2010 and 2011 are adjusted to account for the incomplete coverage of the NNRD during those years; from 2012 onwards the NNRD has complete population coverage of neonatal admissions in England so no adjustment was necessary and data are presented as a rate (95% CI)

Year	Infants recorded in the NNRD	Live births in England	Infants with brain injury to before exclusions	Exclusions	Infants with brain injury to after exclusions	Infants with brain injury adjusted for incomplete NNRD coverage	Infants with brain injury adjusted for incomplete NNRD coverage to after exclusions	Rate of brain injuries per 1000 live births	Brain injuries per 1000 live births to after exclusions (95% CI)
2010	64 375	687 007	2594	59	2535	2743 to 3202	2660 to 3051	3.99 to 4.66	3.87 to 4.44
2011	72 678	688 120	2906	65	2841	2963 to 3159	2889 to 3055	4.31 to 4.59	4.20 to 4.44
2012	78 980	694 241	2950	76	2874	not adjusted	not adjusted	4.25 (4.10,4.41)	4.14 (3.99, 4.29)
2013	80 222	664 517	2966	62	2904	not adjusted	not adjusted	4.46 (4.31,4.63)	4.37 (4.21, 4.53)
2014	85 013	661 496	3097	56	3041	not adjusted	not adjusted	4.68 (4.52,4.85)	4.60 (4.44, 4.76)
2015	88 931	664 399	3055	54	3001	not adjusted	not adjusted	4.60 (4.44,4.76)	4.52 (4.36, 4.68)

‘The annual rate of brain injuries among term infants (≥37 gestational weeks) in England in 2015 was 2.91 (95% CI 2.78 to 3.05) per 1000 live term births; data for term infants born over the period 2010 to 2015 are presented in table 2. The annual rate of brain injuries among preterm infants (<37 gestational weeks) in England in 2015 was 24.45 (95% CI 23.12 to 25.85) per 1000 live preterm births; data for preterm infants between 2010 and 2015 can be found in table 3.’

**Table 2:** Term (≥37 gestational weeks) infants in England with a diagnosis of brain injury to before and after exclusion of conditions leading to brain injury prior to birth; data for 2010 and 2011 are adjusted to account for the incomplete coverage of the NNRD during these years; from 2012 onwards the NNRD has complete population coverage of neonatal admissions in England so no adjustment was necessary and data are presented as a rate (95% CI)

Year	Term infants recorded in the NNRD	Term live births in England	Term infants with brain injury to before exclusions	Term infants with brain injury adjusted for incomplete NNRD coverage	Term infants with brain injury to after adjustment and exclusions	Rate of brain injuries per 1000 term live births to after exclusions (95% CI)
2010	35 415	627 357	1478	1597 to 1795	1562 to 1756	2.49 to 2.80
2011	41 429	630 419	1716	1786 to 1845	1740 to 1798	2.76 to 2.85
2012	46 200	640 787	1714	not adjusted	1661	2.59 (2.47, 2.72)
2013	47 935	612 816	1754	not adjusted	1713	2.80 (2.67, 2.93)
2014	51 945	607 972	1848	not adjusted	1804	2.97 (2.83, 3.11)
2015	55 045	609 076	1808	not adjusted	1771	2.91 (2.78, 3.05)

**Table 3:** Preterm (<37 gestational weeks) infants in England with a diagnosis of brain injury to before and after exclusion of conditions leading to brain injury prior to birth; data for 2010 and 2011 are adjusted to account for the incomplete coverage of the NNRD during these years; from 2012 onwards the NNRD has complete population coverage of neonatal admissions in England so no adjustment was necessary and data are presented as a rate (95% CI)

Year	Preterm infants recorded in the NNRD	Preterm live births in England	Preterm infants with brain injury to before exclusions	Preterm infants with brain injury adjusted for incomplete NNRD coverage	Preterm infants with brain injury after adjustment and exclusions	Rate of brain injuries per 1000 preterm live births to after exclusions (95% CI)
2010	28 960	43 928	1116	1203 to 1238	1173 to 1208	26.71 to 27.51
2011	31 249	44 547	1190	1219 to 1234	1197 to 1213	26.88 to 27.22
2012	32 752	49 949	1236	not adjusted	1213	24.28 (22.96, 25.69)
2013	32 264	48 844	1212	not adjusted	1191	24.38 (23.04, 25.81)
2014	33 036	49 379	1249	not adjusted	1237	25.05 (23.69, 26.49)
2015	33 740	50 308	1247	not adjusted	1230	24.45 (23.12, 25.85)

**Table 4:** Infants in England with conditions leading to brain injury at or soon after birth; infants can be diagnosed with more than one condition so the sum of conditions for each year will not match data given in tables 1–3; P/IVH: periventricular/intraventricular haemorrhage, CI: confidence interval

Condition		Year			
		2012	2013	2014	2015
Seizures	Number of cases	1445	1432	1360	1249
	**Rate per 1000 live births** (95% CI)	**2.1** (2.0 to 2.1)	**2.2** (2.1 to 2.3)	**2.1** (2.0 to 2.2)	**1.9** (1.8 to 2.0)
	Number of term cases	1065	1036	1009	919
	Number of preterm cases	378	396	351	330
Intracranial haemorrhage	Number of cases	754	677	689	726
	**Rate per 1000 live births** (95% CI)	**1.1** (1.0 to 1.2)	**1.0** (0.9 to 1.1)	**1.0** (1.0 to 1.1)	**1.1** (1.0 to 1.2)
	Number of term cases	110	94	104	117
	**Rate per 10 000 term births** (95% CI)	**1.7** (1.4 to 2.1)	**1.5** (1.3 to 1.9)	**1.7** (1.4 to 2.1)	**1.9** (1.6 to 2.3)
	Number of preterm cases	644	583	585	609
	Severe P/IVH<32 weeks gestation	483	445	468	452
	**Rate of severe P/IVH per 1000 live births<32 weeks** (95% CI)	**60.4** (55.2 to 66.0)	**57.7** (52.6 to 63.4)	**61.1** (55.8 to 66.9)	**58.3** (53.1 to 63.9)
Perinatal/neonatal stroke	Number of cases	77	100	88	90
	**Rate per 1000 live births** (95% CI)	**0.11** (0.09 to 0.14)	**0.15** (0.12 to 0.18)	**0.13** (0.11 to 0.16)	**0.14** (0.11 to 0.17)
	Number of term cases	64	78	72	76
	Number of preterm cases	13	22	16	14
Hypoxic-ischaemic encephalopathy	Number of cases	1128	1161	1257	1243
	**Rate per 1000 live births** (95% CI)	**1.6** (1.5 to 1.7)	**1.7** (1.6 to 1.9)	**1.9** (1.8 to 2.0)	**1.9** (1.8 to 2.0)
	Number of term cases	944	971	1044	1018
	Number of preterm cases	184	190	213	225
Central nervous system infection	Number of cases	390	452	543	510
	**Rate per 1000 live births** (95% CI)	**0.56** (0.51 to 0.62)	**0.68** (0.62 to 0.75)	**0.82** (0.76 to 0.89)	**0.77** (0.70 to 0.84)
	Number of term cases	205	275	302	304
	Number of preterm cases	185	177	241	206
Bilirubin encephalopathy	Number of cases	8	7	2	4
	**Rate per 100 000 live births** (95% CI)	**1.2** (0.6 to 2.3)	**1.1** (0.5 to 2.2)	**0.3** (0.1 to 1.2)	**0.6** (0.2 to 1.6)
	Number of term cases	6	5	2	4
	Number of preterm cases	2	2	0	0
Cystic periventricular leucomalacia	Number of preterm cases	199	175	171	184
	**Rate per 1000 live births** (95% CI)	**0.3** (0.3 to 0.3)	**0.3** (0.2 to 0.3)	**0.3** (0.2 to 0.3)	**0.3** (0.2 to 0.3)
	Number of cases at <34 weeks gestation	186	175	157	176
	**Rate per 1000 live births<34 weeks gestation** (95% CI)	**12.7** (11.0 to 14.7)	**12.5** (10.7 to 14.4)	**11.3** (9.7 to 13.3)	**12.4** (10.7 to 14.4)

## Discussion

The third paragraph of the Discussion section of the manuscript is corrected to: ‘The novelty of the measure *brain injuries that occur at or soon after birth,* and the source of the data, the NNRD which is formed from routinely recorded clinical information makes it necessary to consider how incidence rates of individual conditions we report compare with other published data. The annual incidence rates for moderate and severe HIE of between 1.6 and 1.9 per 1000 live births are consistent with other reported rates of neonatal encephalopathy of between 0.77–3.8 per 1000 live births in low neonatal mortality regions such as the United Kingdom and the United States of America. When considering neonatal intracranial haemorrhage, published data commonly reported incidence separately for term and preterm infants. A 30-year-old, single centre study from the USA reported a regional incidence of 2.7 per 10 000 live births for symptomatic intracranial haemorrhage in term infants, which is comparable with the population incidence of 1.5 to 1.9 per 10 000 term births that we report. For preterm infants born at 22–31 weeks gestational age, comparable population-level incidence data for intraventricular/periventricular haemorrhage from the national French EPIPAGE cohort are 3.8% for grade 3% and 3.3% for grade four intra/periventricular haemorrhage. In the same gestational age band, we report annual incidence rates between 5.8% and 6.1% for a composite including grades 3 and 4 intraventricular/periventricular haemorrhage. We report annual incidence rates for neonatal or perinatal stroke of between 0.11 and 0.15 per 1000 live births, which is similar to the estimated minimum incidence rate of 0.10 per 1000 live births reported by a prospective, population-based study from Canada. The annual incidence of neonatal central nervous system infection we report of 0.56 to 0.82 per 1000 live births is similarly in agreement with the population-level incidence rate for neonatal meningitis in England and Wales of 0.39 per 1000 live births (1996–1997) reported by the British Paediatric Surveillance Unit (BPSU). Similarly, annual rates of bilirubin encephalopathy reported here of between 0.3 and 1.2 per 100 000 live births are comparable with BPSU population surveillance rates of 0.9 per 100 000 live births (2003–2005). EPIPAGE 2 (2011) report an incidence among 23 to 34 gestational week infants of 1.8% for cystic periventricular leukomalacia; in the same gestational age group, we report annual rates of between 1.13% and 1.27%. Finally population-based studies of neonatal seizures over the last 30 years report incidence rates between 1.8 and 3.5 per 1000 live births, results that are again in keeping with our annual rates of 1.9 to 2.2 per 1000 live births.’

The following 4 tables (tables 1–4) are corrected as below.

