# Supplementary material for: Neonatal brain injuries in England: population-based incidence derived from routinely recorded clinical data held in the National Neonatal Research Database
Source: Arch Dis Child Fetal Neonatal Ed. 2017 Oct 22;103(4):F301–6. doi: 10.1136/archdischild-2017-313707 (PMC6047140; doi:10.1136/archdischild-2017-313707)
Supplement: Supplementary file 1 [file fetalneonatal-2017-313707supp001.docx]

**Supplemental data 1: Brain injury occurring during or shortly after birth for the national maternity ambition: consensus meeting attendees**

**Attendees**

| *Name* | *Organisation* |
| --- | --- |
| Prof. Neena Modi | Imperial College London |
| Dr Chris Gale | Imperial College London |
| Eugene Statnikov | Imperial College London |
| Dr Sabita Uthaya | Imperial College London |
| Richard Colquhoun | Imperial College London |
| Nilum Patel | Department of Health |
| Sarah Hegarty | Department of Health |
| Madeline Percival | Department of Health |
| Hayley Butcher | Department of Health |
| Karen Todd | Department of Health |
| Emily Weston | Department of Health |
| Siobhain McKeigue | Department of Health |
| Dr Matthew Jolly | NHS England |
| James Wallis | NHS England |
| James Walker | Care Quality Commission |
| Tony Kelly | Kent, Surrey, Sussex Academic Health Sciences Network |
| Michelle Upton | NHS Improvement |
| Birte Harlev-Lam | NHS Improvement |
| Katherine Robbins | NHS Digital |
| Prof Marian Knight | National Perinatal Epidemiology Unit, Oxford |
| Dr David Odd | Senior Clinical Lecturer, University of Bristol |
| Dr Dimitrios Siassakos | Consultant Senior Lecturer, University of Bristol |
| Dr Michael Magro | Darzi Fellow, NHS Litigation Authority |
| Prof Nikki Robertson | Professor of Translational Neonatal Medicine, UCL |
| Prof Alan Cameron | RCOG Vice President, Clinical Quality |
| Prof Donald Peebles | Chair of Maternal Fetal Medicine, UCL |
| Dr Karen Luyt | Consultant Senior Lecturer, University of Bristol |
| Dr Paul Clarke | Consultant Neonatologist, Norfolk and Norwich Hospital |
| Mandy Forrester | My Birthplace® project midwife |
| Julie Frohlich | Consultant midwife, Guy's and St Thomas' Hospital |
| Emily Petch | Royal College of Obstetrics and Gynaecology Each Baby Counts |
| Jacqui Dopran | Senior Neonatal Nurse, Homerton Hospital |

The following were unable to attend and provided written responses:

| *Name* | *Organisation* |
| --- | --- |
| Dr Topun Austin | Consultant Neonatologist, University of Cambridge |
| Dr James Boardman | Reader and Honorary consultant in Neonatal Medicine, University of Edinburgh |
| Dr Frances Cowan | Imperial College London |

Apologies were received from the following:

| *Name* | *Organisation* |
| --- | --- |
| Hannah Knight | Royal College of Obstetrics and Gynaecology |
| Jenny Kurinczuk | National Perinatal Epdiemiology Unit, Oxford |
| Tim Draycott | University of Bristol |
| Prof Neil Marlow | Professor of Neonatal Medicine UCL Institute for Women's Health |
| Prof Marianne Thoresen | Professor of Neonatal Neuroscience, University of Bristol |
| Professor Helen Budge | Professor of Neonatal Medicine, University of Nottingham |
