# Supplementary material for: Neonatal brain injuries in England: population-based incidence derived from routinely recorded clinical data held in the National Neonatal Research Database
Source: Arch Dis Child Fetal Neonatal Ed. 2017 Oct 22;103(4):F301–6. doi: 10.1136/archdischild-2017-313707 (PMC6047140; doi:10.1136/archdischild-2017-313707)
Supplement: Supplementary file 2 [file fetalneonatal-2017-313707supp002.docx]

**Final brain injury definition: National Neonatal Research Database (NNRD) data fields**

**Scope**

- All babies admitted to a NHS neonatal unit in England
- Injury detected during neonatal unit stay to discharge
- Annual data, from January 1^st^ 2010 to December 31^st^ 2015

**Data source**

- Data will be extracted from the National Neonatal Research Database (NNRD) at the Neonatal Data Analysis Unit at Imperial College London.
- The NNRD contains a predefined set of variables (the Neonatal Data Set, an authorised NHS Information Standard) extracted at regular intervals from the Electronic Patient Record of every admission to a NHS neonatal unit in England, Wales, and Scotland, cleaned and merged across multiple patient episodes, to create a single data file for each patient.

| **Condition** | **Data items** |
| --- | --- |
| HIE | Any of the following recorded in any *Diagnosis* field:   1. Severe Hypoxic Ischaemic Encephalopathy (HIE) 2. Severe Neonatal Encephalopathy 3. Grade 3 Hypoxic Ischaemic Encephalopathy (HIE) 4. Moderate Hypoxic Ischaemic Encephalopathy (HIE) 5. Moderate Neonatal Encephalopathy 6. Grade 2 Hypoxic Ischaemic Encephalopathy (HIE)   **OR**  The following recorded in *daily care neurology* field:   1. Therapeutic hypothermia induced - for 2 or more consecutive days |
| Intracranial haemorrhage | Any of the following recorded in any *Diagnosis* field:   1. Subdural haemorrhage due to birth injury 2. Cerebral haemorrhage due to birth injury 3. Traumatic intraventricular haemorrhage 4. Subarachnoid haemorrhage due to birth injury 5. Subarachnoid haemorrhage 6. Tentorial tear due to birth injury 7. Intracranial laceration and haemorrhage due to birth injury 8. Large intraventricular haemorrhage (IVH Grade 3) 9. Intraventricular haemorrhage/parenchymal 10. Parenchymal haemorrhage 11. haemorrhage (IVH Grade 4) 12. Intracranial Haemorrhage (unknown or unspecified cause) 13. Intracerebral haemorrhage 14. Intracerebral haemorrhage (term infant) 15. Intraventricular haemorrhage (perinatal) 16. Post-haemorrhagic hydrocephalus   **OR**  Any of the following recorded in any *cranial ultrasound findings* field:   1. Large intraventricular haemorrhage (IVH Grade 3) 2. Intraventricular haemorrhage/parenchymal haemorrhage (IVH Grade 4) 3. Parenchymal haemorrhage   **OR**  Any of the following recorded in any *procedure field*:   1. Ventriculoperitoneal or other ventricular shunt 2. External ventricular drain 3. Ventricular drain with reservoir 4. Insertion of ventricular peritoneal shunt. 5. Insertion of Rickham reservoir 6. Insertion of ventriculo-atrial CSF shunt 7. Insertion of ventriculo-peritoneal CSF shunt 8. Creation of ventriculoperitoneal shunt |
| Preterm white matter injury | Any of the following recorded in any *Diagnosis* field:   1. Cystic periventricular leukomalacia   **OR**  Any of the following recorded in any *cranial ultrasound findings* field:   1. Cystic periventricular leucomalacia |
| Perinatal stroke | Any of the following recorded in any *Diagnosis at discharge* field:   1. Neonatal stroke 2. Infarction: Middle cerebral artery (stroke) 3. Cerebrovascular accident (stroke) 4. Cerebral venous thrombosis 5. Neonatal cerebral ischaemia |
| Central nervous system infection | Any of the following diagnosis codes recorded in the *Diagnosis* field:   1. Bacterial meningitis 2. Viral meningitis 3. Meningitis – streptococcal 4. Meningitis – bacterial (specific organism) 5. Meningitis – bacterial (unknown or unspecified organism) 6. Meningitis – Candida 7. Candida encephalitis 8. Congenital herpes infection   Any pathogen recorded in the *suspected infection data* field *Pathogen in CSF* |
| Kernicterus | Any of the following diagnoses recorded in any *Diagnosis* field:   1. Bilirubin encephalopathy (immune) 2. Kernicterus (unspecified or unknown cause) 3. Kernicterus |
| Seizures | Any of the following recorded in any *daily care neurology* field:   1. Seizure occurred |

**Exclusions**

Data will be presented before and after exclusion of infants with **seizures** (condition defined as above) AND the following diagnosis codes recorded in the *Diagnosis* field during their their neonatal unit admission.

| **Condition** | **Data items** |
| --- | --- |
| Congenital encephalopathies | **Episodic variables:** Any of the following diagnosis codes entered into any *Diagnosis* field:   1. Congenital neuropathy (unknown or unspecified cause) 2. Congenital myopathy 3. Mitochondrial myopathy 4. Congenital Central Hypoventilation Syndrome (CCHS) 5. Congenital hypertonia 6. Congenital hypotonia - floppy 7. Benign familial neonatal seizures 8. Inborn error of metabolism (description required) 9. Myotonic dystrophy requiring endotracheal intubation and assisted ventilation 10. Disorder of branch chain amino acid metabolism 11. Disorders of fatty acid metabolism 12. Disorders of fatty acid metabolism: carnitine metabolism 13. Disorder of glycine metabolism 14. Disorder of glycine metabolism: Non ketotic hyperglycinaemia 15. Hyperammonaemia of the newborn 16. Disorders of lysine and hydroxylysine metabolism 17. Disorders of ornithine metabolism 18. Disorders of pyruvate metabolism and gluconeogenesis 19. Disorder of carbohydrate metabolism (unknown or unspecified cause) 20. Down Syndrome (Trisomy 21) 21. Trisomy 21 22. Edwards Syndrome (Trisomy 18) 23. Trisomy 18 24. Patau Syndrome (Trisomy 13) 25. Trisomy 13 |
| Congenital infections | **Episodic variables:** Any of the following diagnosis codes entered into any *Diagnosis* field:   1. Congenital viral disease (specify) 2. Syphilis - latent congenital 3. Congenital rubella syndrome 4. Congenital cytomegalovirus infection 5. Congenital herpes [herpes simplex] infection 6. Other congenital viral diseases 7. Congenital viral disease (unknown or unspecified cause) 8. Congenital toxoplasmosis |
| Congenital brain abnormalities | **Episodic variables:** Any of the following diagnosis codes entered into any *Diagnosis* field:   1. Malformations of aqueduct of Sylvius 2. Atresia of foramina of Magendie and Luschka 3. Atresia of foramina of Magendie and Luschka 4. Other congenital hydrocephalus 5. X linked congenital hydrocephalus 6. Congenital hydrocephalus (unknown or unspecified cause) 7. Congenital hydrocephalus 8. Lissencephaly 9. Vermal agenesis 10. Septum pelucidum absence – congenital 11. Congenital malformations of corpus callosum 12. Arhinencephaly 13. Holoprosencephaly 14. Other reduction deformities of brain 15. Septo-optic dysplasia 16. Other specified congenital malformations of brain 17. Congenital malformation of brain (unknown or unspecified cause) 18. Other congenital malformations of brain 19. Cervical spina bifida with hydrocephalus 20. Thoracic spina bifida with hydrocephalus 21. Spina bifida (unknown or unspecified cause) 22. Spina bifida 23. Amyelia 24. Hypoplasia and dysplasia of spinal cord 25. Other specified congenital malformations of spinal cord 26. Congenital malformation of spinal cord (unknown or unspecified cause) 27. Nerve palsies – congenital 28. Arnold-Chiari syndrome 29. Other specified congenital malformations of nervous system 30. Congenital malformation of nervous system (unknown or unspecified cause) 31. Other congenital malformations of nervous system 32. Congenital Hydrocephalus |

**Authors:** Chris Gale, Sabita Uthaya, Eugene Stanikov, Neena Modi, on behalf of the DH expert working group on “*Brain injury occurring during or soon after birth”,* Neonatal Data Analysis Unit

**Date of production:** February 21^st^ 2017
